# PKR downregulation prevents copper-induced synaptic dysfunction and cognitive impairment in a murine model of Wilson’s disease

**DOI:** 10.3389/fnins.2024.1447304

**Published:** 2024-11-25

**Authors:** Chenchen Xu, Songyang Liu, Nan Cheng, Yongsheng Han, Xinheng Wang

**Affiliations:** ^1^Institute of Neurology, Anhui University of Chinese Medicine, Hefei, China; ^2^The Affiliated Hospital of Institute of Neurology, Anhui University of Chinese Medicine, Hefei, China; ^3^Center for Xin’an Medicine and Modernization of Traditional Chinese Medicine of IHM, Anhui University of Chinese Medicine, Hefei, China

**Keywords:** copper, Wilson’s disease, cognitive dysfunction, synapse, oxidative stress, PKR/eIF2α

## Abstract

Synaptic efficacy is critical for memory formation and consolidation. Accumulating evidence suggest that synapses are impaired during Wilson’s disease (WD), contributing to neuronal dysfunction and cognitive decline. WD is a prototypical condition among the copper metabolism disorders. Cognitive impairment is a common feature of affected patients with neurological symptoms, presenting as memory deficits, decreased cognitive flexibility, and impaired learning capabilities. These cognitive deficits can significantly impact the quality of life, affecting work and academic performance. However, the mechanisms mediating the inhibitory synaptic dysfunction in WD are incompletely understood. We investigated the effects of the double-stranded RNA-dependent protein kinase/eukaryotic initiation factor 2α (PKR/eIF2α) pathway on synaptic structure and function in WD using a murine model, toxic milk (TX mice). During mouse open-field tests, we noted a substantial rise in the mobility/immobility ratio among WD model animals compared to that in WT mice. Additionally, WD mice exhibited diminished central area exploration, as evidenced by reduced travel distance. Moreover, they displayed prolonged escape latency in the Barnes maze, suggesting that chronic copper accumulation is associated with neuropsychiatric alterations and cognitive impairment. We also found a decrease in the expression of synapse-associated proteins (synapsin 1, synaptophysin, postsynaptic density protein-93 [PSD93], postsynaptic density protein-95 [PSD95]), and vesicle-associated membrane protein2 [VAMP2]) besides abnormal neurotransmitter levels (including glutamate and GABA), indicating the presence of synaptic dysfunction in TX mice. Inhibiting PKR via C16 prevented these changes, suggesting that dysfunctional cognition is associated with the PKR/eIF2α pathway. We also observed changes in synapses, vesicles, dendritic spine density, and dendritic length that were associated with the presence of cognitive dysfunction. Further investigation revealed that C16 treatment decreased the TUNEL-positive cell numbers in the hippocampus of TX mice and prevented 8-OHdG-induced synaptic dysfunction. Results suggest that PKR downregulation prevents copper-induced synaptic dysfunction in the murine WD model. Therefore, targeting PKR pharmacologically may be a potential therapeutic strategy for treating the copper-induced neuropathology of patients with WD.

## 1 Introduction

Copper is an indispensable trace metal element with a crucial role in the proper functioning of the central nervous system (CNS). Environmental or genetic factors causing copper deficiency or deposition *in vivo* can lead to related CNS damage (Tsang et al., 2021; Jomova et al., 2022). Copper homeostasis dysregulation in the CNS is associated with neurodegenerative disorders such as Wilson’s disease (WD) and Alzheimer’s disease (AD) (Maung et al., 2021; An et al., 2022; Hureau, 2023). WD is an archetypal disease for copper metabolism disorders, with some patients presenting significant cognitive dysfunction that diminishes their health-related quality of life (Rodriguez-Castro et al., 2015; Palmieri et al., 2022). Moreover, copper brain deposition exacerbates the secondary degeneration of cortical neurons induced by nerve injury (Banci et al., 2010). Synaptic dysfunction represents a prominent pathogenic mechanism common to neurodegenerative diseases and cognitive disorders (Chung et al., 2015). Studies in C57BL/6 mice have revealed that excessive copper deposition or heightened copper levels can induce downregulation of synaptic protein expressions, impair synaptic function, and ultimately contribute to cognitive performance decline (Ma et al., 2015).

Double-stranded RNA-dependent protein kinase (PKR) is a ubiquitinated protein that can be activated by various stimuli such as viruses, interferons, endoplasmic reticulum (ER) stress, and reactive oxygen species (ROS) (Hugon and Paquet, 2021). The kinase is a crucial regulator of mRNA translation, cell growth, and apoptosis, as well as a participant of metabolic processes and inflammatory responses, reflecting its involvement in a wide array of physiological and pathological processes (Gal-Ben-Ari et al., 2019). Accumulation of excessive copper in the brain can stimulate the release of reactive oxygen species (ROS), triggering oxidative stress damage (Greenough et al., 2013) and activating the PKR/ eukaryotic initiation factor 2α (eIF2α) pathway (Mouton-Liger et al., 2012; Hirata et al., 2019). PKR, which induces eIF2α phosphorylation and inhibits protein translocations, can negatively modulate the memory function by regulating synaptic plasticity through phosphorylated (*p-*)eIF2α. *p*-eIF2α activates the transcription factor 4 (ATF-4) and reduces the cAMP response elements binding protein (CREB) expression, contributing to cognitive impairments. Moreover, ATF-4 activates the pro-apoptotic molecule C/EBP homologous protein (CHOP), leading to neuronal apoptosis (Pal and Prasad, 2015; Ulrich et al., 2014). Ma’s investigation discovered significantly elevated expression of the oxidative stress marker protein 8-hydroxydeoxyguanosine (8-OHdG) in the brains of C57BL/6 mice due to chronic copper accumulation (Ma et al., 2015). Such accumulation triggers production of PKR, eIF2α, and ATF-4 in the brain, represses CREB activity, and increases CHOP expression. These events result in neuronal apoptosis, decrease synapse-related protein levels (synapsin1, synaptophysin, postsynaptic density protein-93 [PSD93], and postsynaptic density protein-95 [PSD95]), and ultimately lead to cognitive impairment in C57BL/6 mice. Excess copper accumulation in the brain triggers oxidative stress, activates the PKR/eIF2α pathway, and impairs synaptic function, potentially explaining the cognitive dysfunction observed in neurological patients with WD.

Neuroscience research has uncovered an association between synaptic dysfunction (and its regulatory mechanisms) and the development of various neurodegenerative diseases (Chung et al., 2015). These factors may contribute to the synaptic function impairment observed in individuals with WD. Our aim with this study was to explore the possibility that synaptic dysfunction is the cause of the cognitive impairment observed in some individuals with neurological WD. Due to ethical constraints and regulatory limitations of medical clinical trials, conducting research on patients with WD is challenging. Therefore, utilizing appropriate animal models for research purposes is more practical. Common animal models for WD research include the LEC rat, which exhibits a similar WD genetic background as that in humans. Although the LEC rat lacks neurological symptoms or the characteristic K-F rings, it is predisposed to liver cancer. The TX mice, carrying a spontaneous recessive point mutation causing a G712D missense mutation in *Atp7b*, closely resemble the genetic background and biochemical changes of WD, with the mice displaying mild cognitive and motor impairments and slow disease progression. Additionally, *Atp7b*^–/–^ mice, characterized by a well-defined genetic background, exhibit early-stage liver injury but less pronounced neurological symptoms (Dong et al., 2024). Herein, we used a TX mouse model of WD to verify whether the PKR inhibitor C16 can regulate the PKR/eIF2α pathway, prevent copper-induced synaptic dysfunction, and serve as a neuroprotective agent to improve cognitive function in WD mice with cognitive impairment. We hypothesized that by targeting the PKR/eIF2α pathway we would be able to normalize the synaptic function, thereby laying a theoretical foundation for the treatment of cognitive dysfunction in neurological patients with WD. Our experiments assessed the effectiveness of C16 treatment in mice as a means for addressing synaptic dysfunction and restoring cognitive function.

## 2 Materials and methods

### 2.1 Animals

Male and female C3HeB/FeJ (WT) and C3HeB/FeJAtp7btx-J/J (TX) mice were purchased from the Jackson Laboratory (Bar Harbor, ME, USA). We housed all mice at the Neurology Institute, Anhui University of Chinese Medicine facility under standard laboratory conditions (12-h light/dark cycles, 20–22 °C, 50–60% humidity), with *ad libitum* access to water and food throughout the study duration. At 4 weeks of age, mouse tails measuring 1–2 mm were collected and DNA templates were extracted using the Mouse Tail identification kit (YK-MG-100, Ubigene Biosciences). The primer sequences for TX mice were designed as follows: Forward 5’-AGAGTGTGTGCCCTCAGTATCAAAT-3′, Reverse: 5′-TCAAAGAAGGTCACGGGGC-3′. Subsequent to PCR amplification and DNA sequencing, we identified and propagated homozygous TX mice of both sexes. Experiments were conducted using 5-month-old male mice, comprising 63 WT mice and 63 TX mice, distributed as 33 WT mice, 33 TX mice, 33 WT+Saline mice, 15 WT+C16 mice, 15 TX+Saline mice, 15 TX+C16 mice. The Animal Ethics Committees of Anhui University of Chinese Medicine approved all animal experiments.

### 2.2 Drug administration

To inhibit PKR phosphorylation, we administered the PKR inhibitor PKR-IN-C16 (C16, Imidazolo-oxindole PKR inhibitor C16) (Selleck, USA) to WT or TX mice via intraperitoneal injections every morning for 30 days at a dose of 0.2 mL/mouse (300 μg/kg). An equivalent amount of normal saline was administered to the control animals.

### 2.3 Inductively coupled plasma mass spectrometry (ICP-MS)

First, we weighed the hippocampal tissues, then we mixed them with nitric acid and ultrapure water in a 1:1 ratio. After pre-digesting the mixture for 30 min, we transferred it to a microwave digestion apparatus for further digestion. Subsequently, we diluted the solution with ultrapure water and used ICP-MS to quantify the copper levels in the mouse hippocampal tissue.

### 2.4 Open-field tests

We constructed an open-field test apparatus using a 50 cm long, 50 cm wide, and 40 cm high experimental box divided into two zones: the outer zone (zone 1) and the central zone (zone 2). The open field environment was cleared a day prior to the experiment, and mice were acclimated to the room to reduce fear and maintain a quiet setting. On the day of the experiment, we placed each mouse in a predetermined position within the open field box and took measurements for total distance traveled, velocity, distance covered in the central zone, and mobility/immobility ratios. After each trial, we cleaned the box with 75% ethanol to prevent residual odor interference.

### 2.5 Barnes maze test

The Barnes maze test was designed to assess the spatial memory of rodents (Bach et al., 1995). We conducted the test using a 99-cm high circular platform (122-cm of diameter) with 20 evenly-distributed 10-cm diameter holes located 2 cm from the perimeter of the platform and a single escape tunnel mounted on one of the holes. Each mouse was placed in the center of the circular platform (the direction of the mouse’s head was randomly chosen). We recoded a video of each mouse exploring and finding the target escape hole on the platform and measured the escape latency times. We repeated training experiments once a day for 4 days at the same times, and we ran the formal test on the fifth day. The room was kept quiet during the experiments, and the surface of the platform was cleaned with 75% ethanol after each experiment to eliminate olfactory cues in the box.

### 2.6 Spectrophotometric method

We quantified superoxide dismutase (SOD), malondialdehyde (MDA), reduced glutathione (GSH), glutathione disulfide (GSSG), glutamate dehydrogenase (GDH), and glutamate synthase (GOGAT) levels in the hippocampal tissues of mice using spectrophotometry. After extracting the hippocampi, we weighed 0.02-g tissue samples and homogenized them with liquid nitrogen in 200 μL of reagent I from the Solarbio kit (China). Next, we centrifuged the samples at 8000 *g* for 10 min at 4°C. The resulting supernatants were used for analyses. The levels of the aforementioned compounds in each sample were determined using a microplate reader at specific wavelengths as per the manufacturer’s protocol.

### 2.7 Liquid chromatography-mass spectrometry (LC/MS)

We quantified the levels of glutamate (Glu) and gamma-aminobutyric acid (GABA) using LC-MS. We recorded each mouse hippocampus weight and then mixed each sample with 300 μL of methanol containing 0.1% formic acid. The resulting mixtures underwent vortexing for 1 min and homogenization for 5 min. Subsequently, after centrifugation at 14000 rpm for 10 min, we isolated the supernatants and subjected them to analysis via sample injection, following a 20-fold dilution. A standard curve was constructed using standard material with predefined values. We collected the chromatograms of the compounds and integrated them using Xcalibur version 3.0 software, and a linear regression was generated with a weighting coefficient of 1/X2.

### 2.8 Golgi-cox staining

Application of the Golgi-Cox staining technique enables the quantification of neuronal intersections, overall dendritic length, and hippocampal dendrites of mice. After removing the brains of mice, we fixed them with 4% paraformaldehyde for over 48 h. After coronal sectioning of the brain tissues into 2–3-mm slices through the area of interest, we fully submerged each slice in a Golgi staining solution (Service bio, China). These preparations were stored in a cool dry place away from light for 14 days. Afterward, the tissues were treated with 80% glacial acetic acid and 30% sucrose. After further slicing the tissues into 100-μm pieces, we affixed them onto glass slides that had been coated with a mixture of gelatin and potassium chromic sulfate. After examining the samples under a microscope, we used a digital slice scanner (3D HISTECH, Pannoramic250, Hungary) with panoramic multi-layer scanning capabilities to collect the images and analyze them, producing panoramic images of brain tissue.

### 2.9 Transmission electron microscope

Within 1–3 min after coronal sectioning of the brain tissues through the region of interest into 1-mm^3^ slices, we immersed them in electron microscope fixative (2.5% glutaraldehyde) and left them there overnight at 4°C. The samples were then exposed to 1% osmic acid in 0.1 M phosphate buffer PB (PH7.4) for 2 h at room temperature, away from light. Afterward, the specimens were dried at room temperature, encased, solidified in an oven set at 60°C for 2 days, and sliced into 60–80 nm-thick slices ready to be tinted. We examined the samples using a transmission electron microscope (HITACHI, HT7800/HT7700, Japan).

### 2.10 Fluorescent staining

The process of fluorescent staining was carried out in the following manner: After applying transcardial perfusion, we fixed the brains of mice overnight using 4% paraformaldehyde. After coronal sectioning of the brain tissues into 20–30-μm slices, we blocked them with 3% bovine serum albumin (BSA) for 30 min. Next, we incubated the sections with anti-8-OHdG antibody (1:50, Abcam) at 4°C overnight. The next day, we washed the sections three times in PBS and then exposed them to secondary antibodies labeled with fluorescent markers. The incubation process was carried out in the dark at 25 ± 1°C for 1 h. We used a NIKON ECLIPSE C1 microscope to visualize the fluorescence in the samples.

### 2.11 TUNEL staining

We determined the numbers of neuronal cells undergoing apoptosis in the hippocampi of mice using the Fluorescein TUNEL Cell Apoptosis Detection Kit (Service bio, China) in accordance with the manufacturer’s instructions and previously established protocols. An experienced pathologist unaware of the experimental design used a BX53 microscope (Olympus) to visualize the samples.

### 2.12 Quantitative real-time PCR (RT-qPCR)

We extracted total RNA from hippocampal tissues of whole brain samples using the TRIZOL method. Subsequently, cDNA templates were generated via reverse transcription using the SPARKscript ? RT Plus Kit (With gDNA Eraser) (AG0304-B, SparkJade). We set up PCR reactions to assess the relative mRNA levels of *Pkr*, *eIF2alpha*, *Atf4*, *Creb1*, *chop*, *Synapsin-1*, *Syn*, *Psd93*, *Vamp2*, and *Psd95*. Table 1 provides the relevant primer details. The qPCR cycling conditions comprised an initial denaturation at 94°C for 3 min, followed by 40 cycles of 94? for 10 s and 60°C for 34 s. Each sample was analyzed in triplicate, and the results were normalized to beta-actin and the Chow group, presented as the 2^–ΔΔCt^ value.

**TABLE 1:** Primers used for qPCR.

Genes	Primer sequences
*beta-actin*	Forward: 5′-GTGACGTTGACATCCGTAAAGA-3′
	Reverse: 5′-GTAACAGTCCGCCTAGAAGCAC-3′
*Pkr*	Forward: 5′- GTTAAAGAGCCCGCCGAAA-3′
	Reverse: 5′- CAGGTGCTGACTGGGAAACA-3′
*Syn*	Forward: 5′-AGTGGGTCTTTGCCATCTTCG-3′
	Reverse: 5′-CCGAGGAGGAGTAGTCACCAAC-3′
*Atf4*	Forward: 5′-AGACACCGGCAAGGAGGATG-3′
	Reverse: 5′-AAGAGCTCATCTGGCATGGTTT-3′
*eIF2alpha*	Forward: 5′-CCCAGGAAGTAAAGGTGCCC-3′
	Reverse: 5′-GGAGGCTCCTGTCTTGTCAACC-3′
*Psd95*	Forward: 5′-GCAGGTTGCAGATCGGAGAC-3′
	Reverse: 5′-ACTGATCTCATTGTCCAGGTGCT-3′
*Synapsin-1*	Forward: 5′-GATGCTAAATATGATGTGCGTGTC-3′
	Reverse: 5′-AATGTGATCCCTTCCGTCCTT-3′
*chop*	Forward: 5′-CCAGGAAACGAAGAGGAAGAAT-3′
	Reverse: 5′-CACTGACCACTCTGTTTCCGTTT-3′
*Psd93*	Forward: 5′-CGAACCAATCAGAAACGCTCC-3′
	Reverse: 5′-TTCTTTCCACCCTCCGCTTG-3′
*Creb1*	Forward: 5′-TGGCTAACAATGGTACGGATGG-3′
	Reverse: 5′-GTGCTGTGCGGATCTGGTATGT-3′
*Vamp2*	Forward: 5′-GCTGGATGACCGTGCAGAT-3′
	Reverse: 5′-GATGGCGCAGATCACTCCC-3′

### 2.13 Western blotting

After anesthetizing the mice, we excised the hippocampi and added 1 mL of RIPA buffer (R0020, Solarbio) to each 100–130-mg sample of hippocampal tissue. The tissue was homogenized using a high-speed tissue grinder at 60 HZ for 40 s, followed by incubation on ice for 30 min with periodic mixing every 10 min to ensure complete lysis. After centrifugation at 12000 rpm for 30 min at 4°C, we collected the supernatants and added 4*SDS-PAGE loading buffer (with β-Mercaptoethanol) (P1016, Solarbio) to each one. The samples were then boiled in a metal bath at 100°C for 8 min and stored at −80°C. Protein quantifications were performed using the BCA assay. Electrophoresis was carried out using 20-μg protein samples on gels with 8% and 12% separation chosen based on the target proteins’ molecular weights. The gel composition included 30% Acrylamide (A1010, Solarbio), 1.5 M Tris HCL (T1010, Solarbio), 10% SDS (S1010, Solarbio), 10% AP (EC0007, SparkJade), TEMED (T8090, Solarbio), and ddH_2_O. After the protein separation, we transferred the proteins onto nitrocellulose membranes. The membranes were blocked with 5% skim milk powder for 4 h and washed three times with TBST for 10 min each. We incubated the membranes with different antibodies, namely rabbit anti-PKR antibody (1:2000 dilution, ab184257, Abcam), rabbit anti-Phospho-PKR (Thr451) antibody (1:1000 dilution, 44-668G, Invitrogen), rabbit anti-eIF2α antibody (1:1000 dilution, 5324, Cell Signaling Technology), rabbit anti-Phospho-eIF2α (Ser51) antibody (1:1000 dilution, 3398, Cell Signaling Technology), rabbit anti-ATF-4 antibody (1:1000 dilution, 11815, Cell Signaling Technology), rabbit anti-CREB antibody (1:1000 dilution, 9197, Cell Signaling Technology), rabbit anti-Phospho-CREB (Ser133) antibody (1:1000 dilution, 9198, Cell Signaling Technology), rabbit anti-CHOP antibody (1:1000 dilution, 5554, Cell Signaling Technology), rabbit anti-Synapsin1 antibody (1:1000 dilution, 5297, Cell Signaling Technology), rabbit anti-Synaptophysin antibody (1:1000 dilution, 36406, Cell Signaling Technology), rabbit anti-PSD-93 antibody (1:1000 dilution, 19046, Cell Signaling Technology), rabbit anti-PSD-95 antibody (1:1000 dilution, 3450, Cell Signaling Technology), and rabbit anti-VAMP2 antibody (1:1000 dilution, GB11451, Service bio) at 4°C overnight. The next day, after washing the membranes, we added horseradish peroxidase-labeled goat anti-rabbit IgG (1:10000 dilution, ZB2301, ZSGB-BIO) and incubated the solutions in a shaker for 1.5 h. The membranes were again washed three times with TBST for 10 min each. We used the rabbit anti-α-tubulin antibody (1:1000 dilution, R23452, ZENBIO) as an internal control. Finally, we used a gel imaging system to scan and analyze the optical density of each band.

### 2.14 Statistical analyses

We conducted all statistical analyses using GraphPad Prism 8 (GraphPad, La Jolla, CA, USA). All data are expressed as means ± standard errors of the mean. Initially, the Shapiro-Wilk test was conducted on the data, resulting in *P* > 0.05 which indicates normal distribution. Meanwhile, a homogeneity of variance test was also performed. The Brown-Forsythe test was used, and *P* > 0.05 indicated that the homogeneity of variance test passed. Consequently, parametric tests were used for data analysis. We applied Student’s *t*-test to obtain single variant analyses and compared differences between means among multiple groups using one-way or two-way analyses of variance (ANOVAs); we considered *P*-values < 0.05 as statistically significant.

## 3 Results

### 3.1 Copper deposition and behavioral abnormalities in TX mice

**FIGURE 1:**
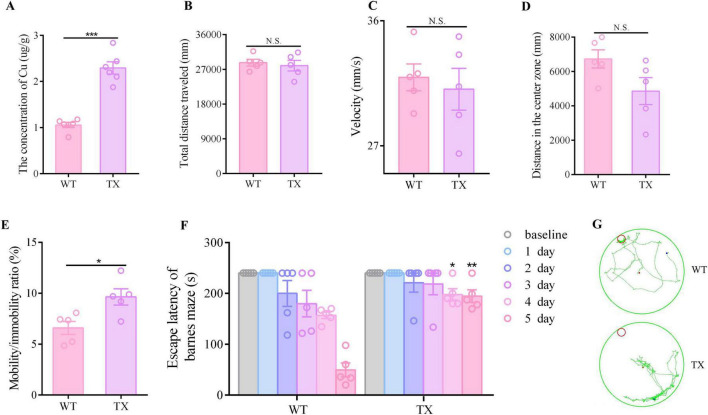
Copper deposition and behavioral abnormalities in the murine model of Wilson’s disease (WD). **(A)** We measured the copper content in the hippocampal tissue of wild-type (WT) and TX mice using ICP-MS (*n* = 6). The open field test was used to observe the neuropsychiatric changes in WT and TX mice, and the total distance traveled **(B)**, velocity **(C)**, distance traveled in the central zone **(D)**, and mobility/immobility ratio **(E)** were recorded (*n* = 5). **(F)** Escape latency in the Barnes maze test (*n* = 5). **(G)** Barnes maze test results showing the trajectory of an individual mouse over a 4 min-testing session (*n* = 5). Numeric values represent means ± SEM; *P < 0.05, **P < 0.01, ***P < 0.001.

The copper content in the hippocampal tissues of wild-type (WT) mice and TX mice were initially quantified using ICP-MS. We observed a significant copper deposition increase in the hippocampi of TX mice compared to that in WT mice (Figure 1A). Subsequently, we compared the behavioral markers in TX mice to those in WT mice to assess the presence of behavioral abnormalities in the WD model mice. The open field test was employed to assess neuropsychiatric changes in both groups, measuring total distance traveled, velocity, distance in the center zone, and mobility/immobility ratio. Our results indicated similar total distances traveled (Figure 1B) and similar velocities (Figure 1C) in TX and WT mice. However, TX mice exhibited reduced distance traveled in the central area compared to WT mice (Figure 1D). Moreover, the mobility/immobility ratios were significantly higher in TX mice than in WT mice (Figure 1E), suggesting increased anxiety and neuropsychiatric changes in TX mice. The Barnes maze test was used to evaluate cognitive impairment, results reveaed a time-dependent decrease in escape latency across all groups. Notably, TX mice displayed prolonged escape latencies on days 4 and 5 compared to the escape latencies in WT mice, with a more pronounced difference on day 5 (Figures 1F, G). These findings are consistent with the presence of copper deposition in TX mice, with behavioral abnormalities indicative of cognitive impairment.

### 3.2 Synapsis impairment in mice with Wilson’s disease (WD)

**FIGURE 2:**
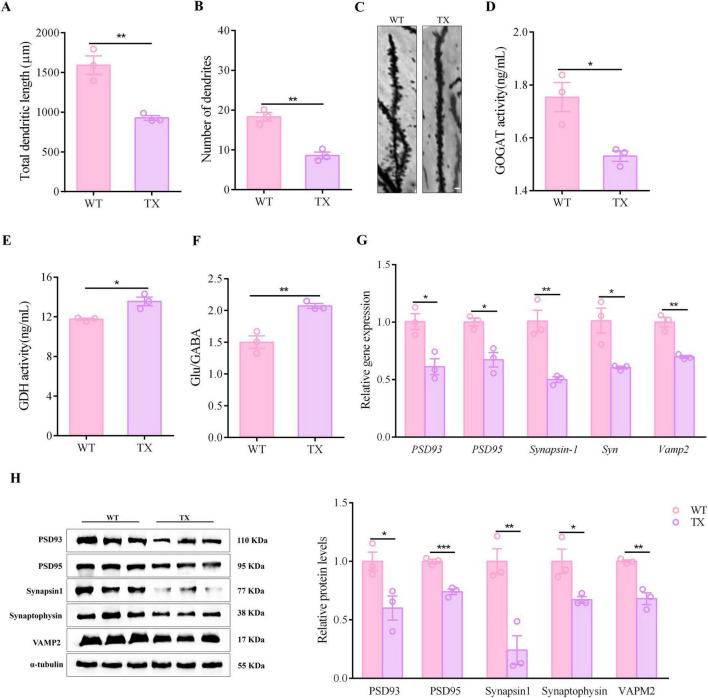
Evidence of synaptic dysfunction in animal model of Wilson’s disease (WD). **(A)** Total dendritic lengths in wild type (WT) and toxic milk (TX) mice (*n* = 3). **(B)** Number of dendrites in TX mice compared to the number in control mice (*n* = 3). **(C)** Images of hippocampal neurons of TX and WT mice. Scale bar, 10 μm. **(D,E)** Microplate reader results showing hippocampal GDH and GOGAT activities (*n* = 3). **(F)** LC/MS quantitative results for glutamate/GABA metabolic loop-related indicators in WT and TX mice (*n* = 3). **(G)** RT-qPCR results for mRNA expressions of *Synapsin-1, Syn* and *Vamp2* in presynaptic membranes, and *PSD93* and *PSD95* in postsynaptic membranes of WT and TX mice (*n* = 3). **(H)** Levels of synapsin1, synaptophysin, and VAMP2 in presynaptic membranes, and of PSD93 and PSD95 in postsynaptic membranes of WT and TX mice (*n* = 3), as quantified using western blots (we normalized the relative protein levels against those of the tubulin loading control and present them as relative protein levels compared to the control). Numeric values represent means ± SEM; *P < 0.05, **P < 0.01, ***P < 0.001.

Neurodegenerative diseases and cognitive dysfunction disorders share synaptic dysfunction as a critical pathogenetic mechanism. Our investigation focused on determining the presence of synaptic dysfunction in WD. Golgi-Cox staining of TX mice hippocampi revealed a significant reduction in total dendritic length and number of dendrites (Figures 2A–C). The mammalian CNS relies heavily on γ-aminobutyric acid (GABA) as its primary inhibitory neurotransmitter, whereas glutamate serves as the main excitatory neurotransmitter. The glutamate/GABA metabolic loop is the primary pathway for metabolic transition between these two neurotransmitters in the brain. Compared to their wild-type counterparts, TX mice exhibited a decrease in glutamate synthase GOGAT activity (as shown in Figure 2D), whereas glutamate dehydrogenase (GDH) activity and the ratio of glutamate to GABA were increased (Figures 2E, F). The data we collected indicate that TX mice experienced a disruption in their excitatory and inhibitory signaling, resulting in an overabundance of possible excitotoxicity. This process may have further triggered oxidative stress and ultimately hindered the proper functioning of their synapses. In the hippocampus of TX mice, we found a reduction in the mRNA levels of *Synapsin-1, Syn* and *Vamp2* in the presynaptic membrane, as well as reduced mRNA levels of *Psd93* and *Psd95* in the postsynaptic membrane, when compared to the levels in WT mice (Figure 2G). Moreover, we observed decreased levels of synapsin1, synaptophysin, VAMP2, PSD93, and PSD95 proteins in the hippocampus of TX mice (Figure 2H). These results are indicative of synaptic dysfunction in TX mice.

### 3.3 Oxidative stress damage of the hippocampus in TX mice

**FIGURE 3:**
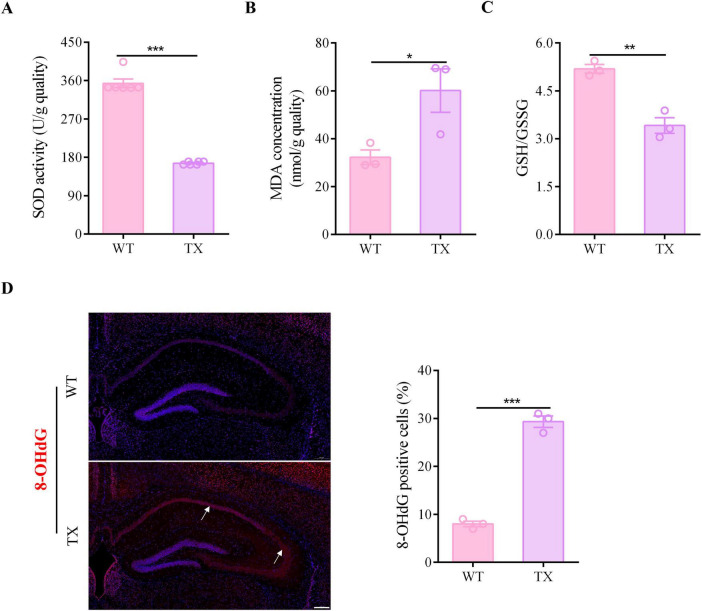
Oxidative stress damage in murine model of Wilson’s disease (WD). **(A)** Levels of superoxide dismutase (SOD) in the hippocampus of wild type (WT) and toxic milk (TX) mice quantified using a microplate reader (*n* = 6). **(B)** Levels of malondialdehyde (MDA) in the hippocampus of WT and TX mice (*n* = 3). **(C)** Microplate reader-detected levels of GSH/GSSG in the hippocampus of WT and TX mice (*n* = 3). **(D)** Immunofluorescence staining (red) and quantification of 8-Hydroxydeoxyguanosine (8-OHdG) in the hippocampi of WT and TX mice. 8-OHdG positive cells are indicated with an arrow. Scale bar, 200 μm (*n* = 3). Numeric values represent means ± SEM; *P < 0.05, **P < 0.01, ***P < 0.001.

We measured the levels of SOD, MDA, GSH, and GSSG in the hippocampus of both WT and TX mice using a microplate reader to verify the presence of oxidative stress damage in our WD model mice. Moreover, we labeled tissues and quantified the presence of 8-OHdG, a marker for oxidative stress, by immunofluorescence staining. TX mice exhibited lower SOD activity levels in the hippocampus than the WT mice (Figure 3A). The MDA content in the hippocampus of TX mice was higher than that of WT mice (Figure 3B). These data indicate that the ratio of GSH to GSSG in the hippocampus of TX mice was lower than that of WT mice (Figure 3C). The number of 8-OHdG positive cells for was significantly higher in the hippocampus of TX mice than in that of WT mice (Figure 3D). Our data indicate that oxidative stress damage is a characteristic feature of TX mice.

### 3.4 Activation of the PKR/eIF2α pathway in TX mice

**FIGURE 4:**
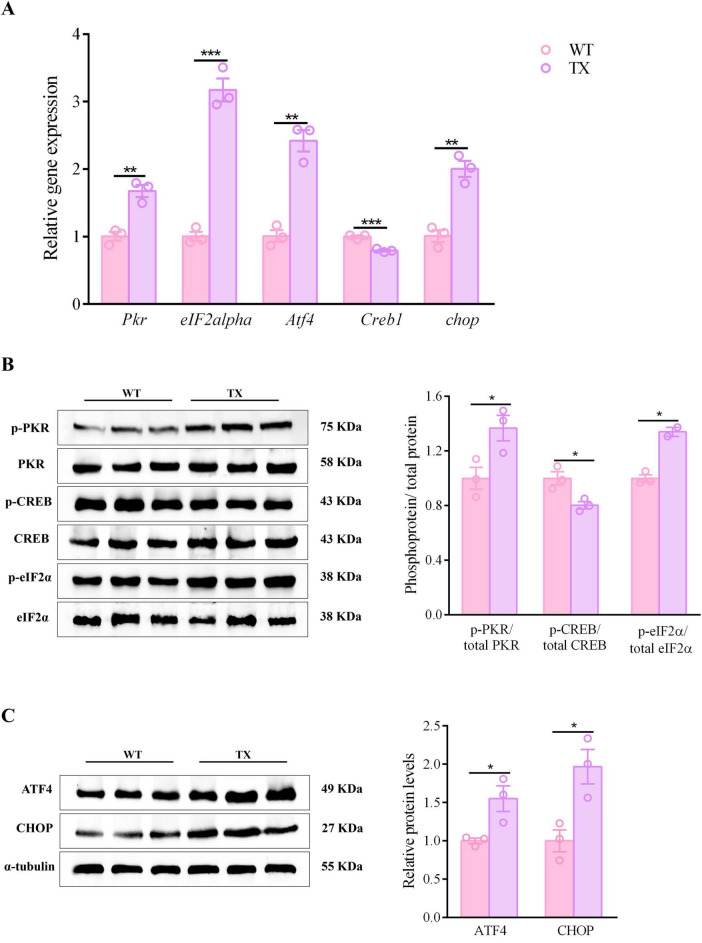
PKR/eif2α pathway activation in murine model of Wilson’s disease (WD). **(A)** Quantification of mRNA expression levels of *Pkr*, *eIF2alpha*, *Atf4*, *Creb1*, and *chop* in the hippocampus of wild type (WT) and toxic milk (TX) mice using quantitative real-time PCR (RT-qPCR) (*n* = 3). **(B,C)** Levels of *p*-PKR/total PKR, *p*-eIF2α/total eIF2α, ATF4, *p*-CREB/total CREB, and CHOP quantified using western blotting in WT and TX mice (*n* = 3) (we normalized the relative protein levels against those of the tubulin loading control and present them as relative protein levels compared to the control). Numeric values represent means ± SEM; *P < 0.05, **P < 0.01, ***P < 0.001.

Synaptic dysfunction plays a significant role in the development of CNS diseases. The cognitive impairment observed in neurological patients with WD may be due to synaptic dysfunction caused by copper. Evidence suggests that prolonged exposure to copper may trigger the PKR/eIF2α signaling pathway, decreasing the expression of synaptic proteins, resulting in synaptic dysfunction and memory impairment. Our investigation aimed to determine whether TX mice display PKR/eIF2α pathway activation. Compared to their wild-type counterparts, TX mice exhibited increased mRNA levels of *Pkr*, *eIF2alpha*, *Atf4*, and *chop* in their hippocampi, as determined by RT-qPCR analysis, while their expression of *Creb1* was decreased (Figure 4A). We found that the levels of phosphorylated proteins PKR and eIF2α were higher in TX mice than in WT mice according to western blot results, whereas the ratio of total CREB to p-CREB was lower in TX mice than in WT mice (Figure 4B). Western blotting showed higher ATF4 and CHOP protein expressions in TX mice than in WT mice (Figure 4C). TX mice exhibited both protein and mRNA synaptic dysfunction, as evidenced by RT-qPCR and western blot findings.

### 3.5 C16 protection against behavior impairment and hippocampal neuronal damage attenuation in murine model of Wilson’s disease (WD)

**FIGURE 5:**
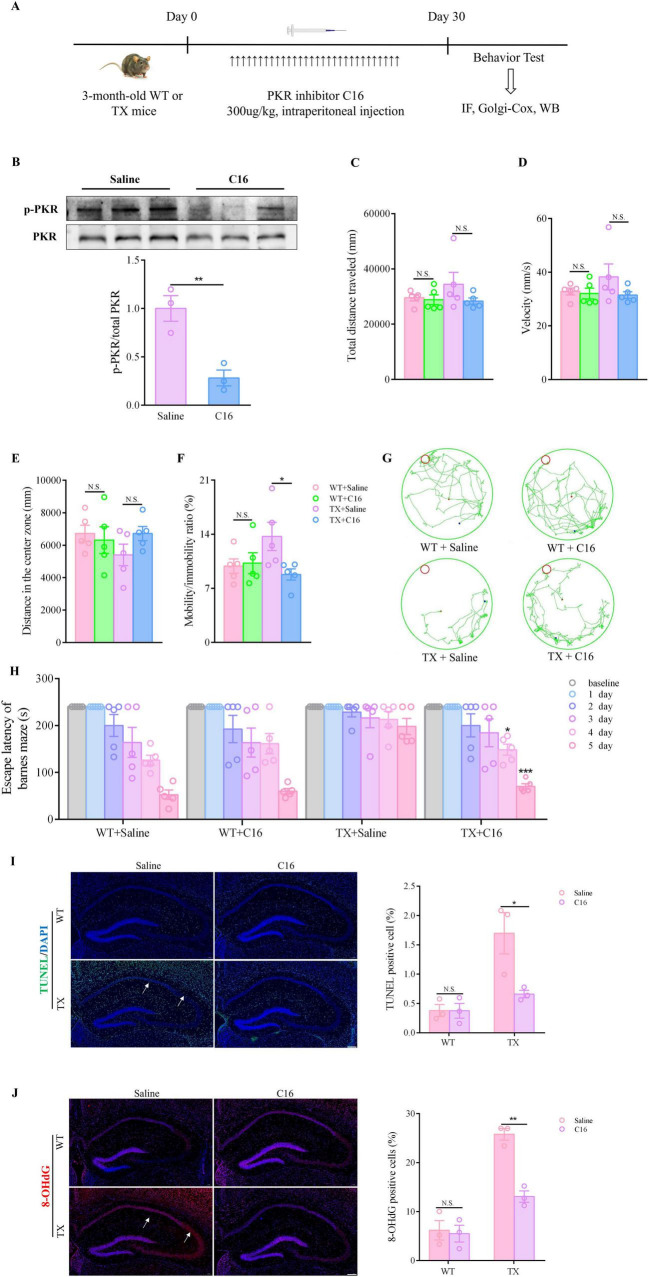
C16, a PKR inhibitor, provides protection against behavior impairment and reduces hippocampal neuronal damage in murine model of Wilson’s disease (WD). **(A)** Flow chart of PKR inhibitor C16 treatment. **(B)** Western blot results showing total PKR and phosphorylated PKR in the hippocampus of mice and *p*-PKR: total PKR ratio (*n* = 3); the toxic milk (TX) mice were either treated with the C16 PKR inhibitor or an equivalent mock treatment. We used open field tests to observe the neuropsychiatric changes in WT or TX mice treated with C16 or saline for 30 days (*n* = 5): **(C)** total distance traveled, **(D)** velocity, **(E)** distance traveled in the center zone, and **(F)** mobility/immobility ratio. **(G)** Escape latency in the Barnes maze test (*n* = 5). **(H)** Barnes maze test results showing the trajectory of an individual mouse over a 4 min-testing session (*n* = 5). **(I)** TUNEL (green) immunofluorescence staining showing apoptotic neurons of WT or TX mice treated with C16 or saline for 30 days. The arrows point to apoptotic neurons. Scale bar, 200 μm (*n* = 3). **(J)** Immunofluorescence staining (red) and quantification of 8-hydroxydeoxyguanosine (8-OHdG) in the hippocampus of WT and TX mice treated with C16 or saline for 30 days. The arrows point to 8-OHdG positive cells. Scale bar, 200 μm (*n* = 3). Numeric values represent means ± SEM; *P < 0.05, **P < 0.01, ***P < 0.001.

We tested the effectiveness of C16, a strong inhibitor of PKR (in TX mice *in vivo*) to investigate whether pharmacologically blocking the activation of the PKR/eIF2α pathway could prevent synaptic dysfunction. TX mice were administered an intraperitoneal injection of C16 to inhibit PKR phosphorylation (Figure 5A). As anticipated, the proportion of *p*-PKR to overall PKR protein decreased in the hippocampus of the C16-treated TX mice (Figure 5B). We also investigated whether administering C16 had any positive impact on the neuropsychiatric changes and cognitive abilities of the mice. Figures 5C, D show the minimal differences observed in total distances traveled and velocities before and after C16 treatments. However, C16-treated TX mice showed increased activity in the central zone during the open-field tests, along with a notable decrease in their mobility/immobility ratios (Figures 5E, F). Additionally, in the Barnes maze task, TX mice displayed significantly reduced escape latencies on days 4 and 5 post-C16 treatment compared to those in untreated TX mice, particularly on day 5 (Figures 5G, H). Thus, the elimination of PKR phosphorylation by C16 shielded TX mice from behavioral impairment and cognitive decline. Moreover, we investigated the effect of C16 administration on hippocampal neurons using immunofluorescence staining. Our findings indicate that C16 administration resulted in a significant reduction in the number of TUNEL-positive cells, compared with the findings in the TX mice that had not receive C16 treatment (Figure 5I). The number of cells in the hippocampus of TX mice that tested positive for 8-OHdG decreased significantly following a 30-day C16 treatment (Figure 5J). According to our data, C16 has a beneficial effect attenuating behavior impairment and neuronal damage in TX mice.

### 3.6 Synaptic dysfunction prevention by PKR inhibitor C16 treatment in murine model of Wilson’s disease (WD)

**FIGURE 6:**
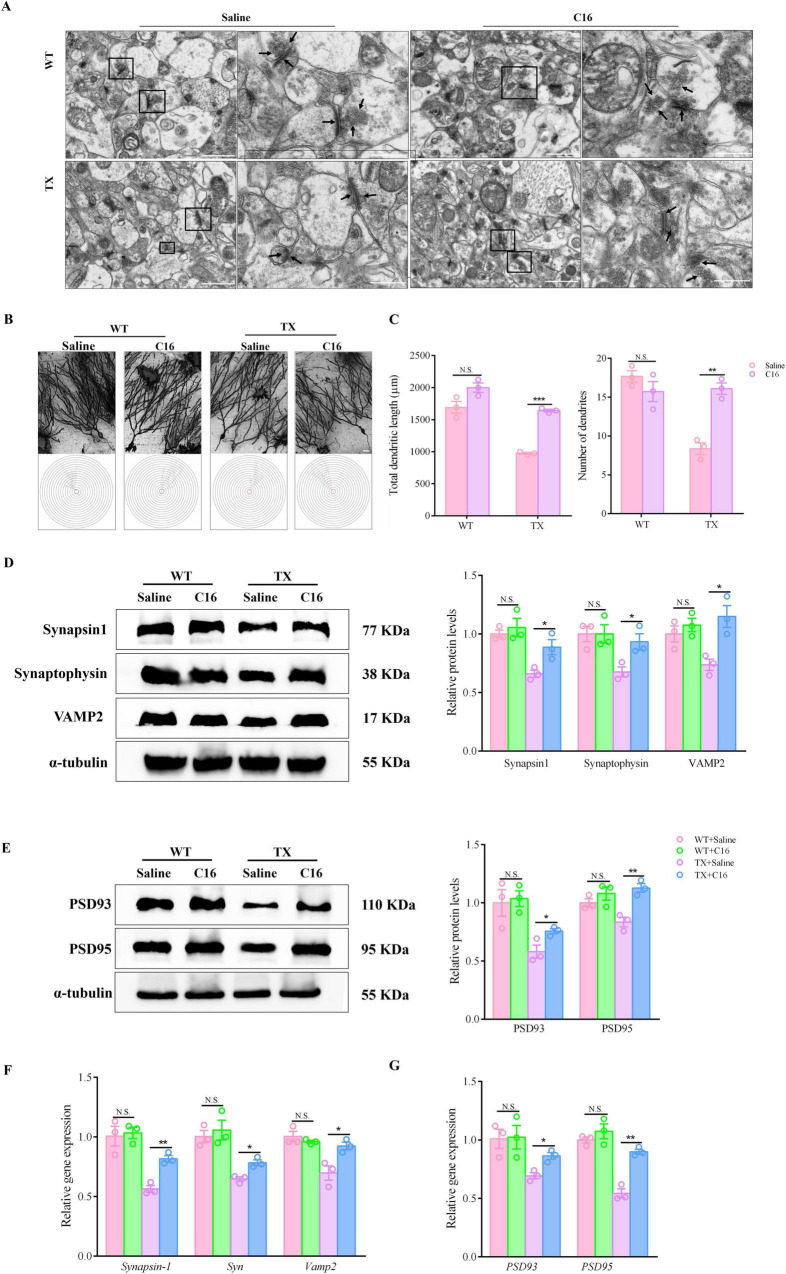
C16, a PKR inhibitor, attenuates the synaptic dysfunction of Wilson’s disease (WD) in murine model. **(A)** Ultrastructural organization of hippocampal organotypic slices in saline- or C16-treated toxic milk (TX) and wild type (WT) mice by transmission electron microscope. The right-side images are enlarged versions of the black boxes on the left images, and the arrow points to synaptic vesicles and the pre- and post-synaptic membranes. Scale bars on the left represent 1 μm, and those on the right represent 500 nm. **(B)** Schematic photomicrographs of neurons with dendrites between repeated 20 mm-spaced concentric rings in the hippocampus of untreated or C16-treated TX and WT mice quantified using Golgi-Cox staining. **(C)** Total dendritic length and number of dendrites in the hippocampus of untreated or C16-treated TX and WT mice quantified using Golgi-Cox staining. **(D,E)** Levels of synapsin1, synaptophysin, VAMP2, PSD93, and PSD95 in the hippocampus of saline- or C16-treated TX and WT mice quantified using western blotting (*n* = 3) (we normalized the relative protein levels against those of the tubulin loading control and present them as relative protein levels compared to the control). **(F,G)** RT-qPCR results for mRNA expressions of *Synapsin-1*, *Syn* and *Vamp*2 in presynaptic membranes, and *PSD93* and *PSD95* in postsynaptic membranes of saline- or C16-treated TX and WT mice (*n* = 3). Numeric values represent means ± SEM; *P < 0.05, **P < 0.01, ***P < 0.001.

Our next step was to determine whether the C16 treatment could reduce synaptic dysfunction. We examined morphological alterations in the hippocampal neurons of mice using a transmission electron microscopy (TEM) and Golgi-Cox staining before and after C16 treatment. These changes primarily involve synapses, vesicles, dendritic spine density, and dendritic length. TEM images indicated that untreated TX mice exhibited decreased synapse counts, fewer pre-synaptic membrane vesicles, shrunken neuronal nuclei, and indistinct pre- and post-synaptic membrane boundaries compared to WT mice. Conversely, C16 treatment restored neurons to normal synapse numbers, uniform vesicle abundances, and well-defined pre- and post-synaptic membranes in both C16-treated and untreated WT mice. TX mice treated with C16 showed increased synapse and synaptic vesicle numbers, along with clear membrane boundaries (Figure 6A). Golgi-Cox staining of TX mice hippocampi revealed reduced total dendritic lengths and dendrite numbers compared to those in WT mice. Following C16 treatment, we noted a rise in neuronal intersections, total dendritic lengths, and dendrite counts in TX mice hippocampi (Figures 6B, C). Moreover, mRNA levels of *Synapsin-1, Syn, Psd93, Vamp2*, and *Psd95* were reduced in the hippocampus of TX mice compared to the levels in WT mice, as well as protein levels. We then examined the discrepancies in the levels of particular synaptic proteins in the pre- and post-synaptic membranes of hippocampal neurons of C16-treated TX mice and found significant increases in the expression levels of synapsin1, synaptophysin, and VAMP2 in the pre-synaptic membrane regions of their hippocampi (Figure 6D), as well as other proteins in the post-synaptic membrane regions, such as PSD93 and PSD95(Figure 6E), as well as mRNA levels (Figures 6F, G), when comparing the levels to those in untreated TX mice. Thus, C16 appears to attenuate the synaptic dysfunction that arises from copper accumulation in TX mice.

### 3.7 PKR/eIF2α pathway activation inhibition by the PKR inhibitor C16 in murine model of Wilson’s disease (WD)

**FIGURE 7:**
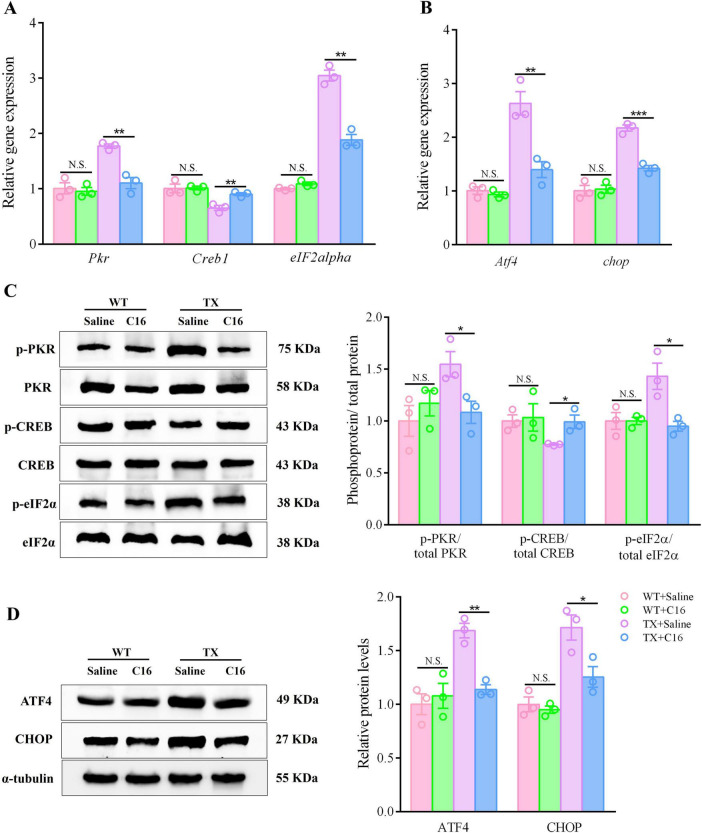
The PKR inhibitor C16 inhibits activation of the PKR/eIF2α pathway in a murine model of Wilson’s disease (WD). **(A,B)** Quantification of mRNA expression levels of *Pkr*, *eIF2alpha*, *Atf4*, *Creb1*, and *chop* in the hippocampus of saline- or C16-treated toxic milk (TX) and wild type (WT) mice using quantitative real-time PCR (RT-qPCR) (*n* = 3). **(C,D)** Levels of *p*-PKR/total PKR, *p*-eIF2α/total eIF2α, ATF4, *p*-CREB/total CREB, and CHOP in the hippocampi of the saline- or the C16-treated toxic milk (TX) and wild type (WT) mice quantified using western blotting (*n* = 3) (we normalized the relative protein levels against those of the tubulin loading control and present them as relative protein levels compared to the control). Numeric values represent means ± SEM; *P < 0.05, **P < 0.01, ***P < 0.001.

Although the underlying mechanisms remain unclear, our data show that C16 was effective in alleviating the cognitive and synaptic dysfunction of TX mice. We investigated whether C16 administration suppressed the PKR/eIF2α pathway *in vivo*, as hypothesized. Following intraperitoneal injections of C16, TX mice showed reduced mRNA levels of *Pkr*, *eIF2alpha*, *Atf4*, and *chop* in their hippocampi, as determined by RT-qPCR analysis. Conversely, the expression of *Creb1* mRNA was upregulated (Figures 7A, B). Meanwhile, Western blot analysis showed that the TX mice exhibited a significant reduction in the expression levels of phosphorylated PKR and eIF2α, while simultaneously revealing an increase in the expression level of phosphorylated CREB in the C16 treatment condition. In addition, C16 treatment reduced the expression of ATF4 and enhanced the expression of CHOP in the hippocampal neurons of TX mice (Figures 7C, D). Our results suggest that the inhibition of the activation of the PKR/eIF2α pathway by the PKR inhibitor C16 attenuated the synaptic dysfunction caused by copper deposition and provided cognitive protection to TX mice.

## 4 Discussion

The association between copper homeostasis and cognitive function has attracted the attention of many researchers. We previously reported that excessive amounts of copper in a copper-overload rat model causes hyperphosphorylation of tau protein in the hippocampal CA1 area of the animals (Xu et al., 2015). This leads to an increase in Aβ1-42 in the CA1 region and cognitive impairment. Animal studies by Zhao et al. (2021) demonstrated that the injection of Cu-Aβ1-42 into the hippocampus of C57BL/6 mice induces cognitive impairment that is ameliorated significantly following treatment with a copper chelator. Our findings indicate that the hippocampal copper levels in WD model TX mice are higher than in WT mice and correlate with behavioral anomalies, particularly cognitive deficits; this finding aligns with prior research findings. Neuronal synapses have crucial physiological roles and participate in various forms of learning. Synaptic dysfunction can lead to cognitive deficits and is a significant contributor to the development of neurodegenerative diseases. The correlation between cognitive function and synaptic protein expression is well-established (Cheng et al., 2021). Studies have revealed a significant reduction in the levels of synaptophysin, synapsin1, and PSD95 in the hippocampus after hypobaric hypoxia, suggesting a decline in memory retention (Kumari et al., 2020). Chronic copper accumulation *in vivo* has been shown to decrease synaptic protein expression, resulting in impaired cognitive function, particularly in spatial memory abilities. This is in line with the findings of a study by Ma et al. (2015) on TX mice, which revealed significant reductions in total dendritic length and dendrite numbers, decreased synapse counts, lower levels of pre-synaptic membrane vesicles, shrunken neuron nuclei, and indistinct pre- and post-synaptic membrane boundaries in the mice with chronic copper accumulation. Moreover, synaptic-related mRNA and protein levels were reduced in TX mice, indicating synaptic dysfunction. Additionally, Tönnies and Trushina (2017) found that chronic copper deposition in the brain led to a significant increase in Aβ levels, which in turn triggered the aggregation of Aβ and subsequent generation of ROS, resulting in cognitive impairment in patients with AD. Our data suggests that oxidative stress damage is a prominent feature in TX mice, as evidenced by the microplate reader analysis results. Moreover, TX mice exhibited disturbances in excitatory and inhibitory signaling, leading to an excess of excitotoxicity, which could further exacerbate oxidative stress and disrupt synaptic function. Our findings underscore the potential for persistent copper accumulation in the brain to induce ROS production, contributing to the synaptic dysfunction and cognitive decline in patients with WD. Thus, synaptic dysfunction may be a characteristic of WD development, leading to cognitive impairment.

WD is an autosomal recessive copper metabolism disorder. Cognitive dysfunction (such as memory loss, insensitivity, decreased learning ability, and problem-solving challenges) is evident in some patients with WD, particularly in those with neurological manifestations. These impairments can diminish their quality of life, impacting their performance in working and academic settings (Rodriguez-Castro et al., 2015). Cognitive impairment in neurological patients with WD is primarily caused by copper accumulation in brain regions associated with cognition. In addition, excessive copper levels can lead to synaptic dysfunction, a potential mechanism in the development of WD. We confirmed the presence of cognitive dysfunction in a murine model of WD with mice displaying behavioral changes and neuronal injury. In addition, we observed synaptic dysfunction in TX mice through Golgi staining, as well as by identification of neurotransmitter contents (such as glutamate and GABA) and levels of synapse-associated proteins. Therefore, studying the mechanisms of synaptic dysfunction induced by copper accumulation is important to develop effective therapeutic interventions to alleviate the cognitive impairment of these patients. At present, the primary treatment for patients with WD involves copper chelating agents, which have a therapeutic benefit for some individuals, but only limited efficacy for those with cognitive impairment and neurological symptoms. Thus, exploring alternative treatments is important. Studies have found that PKR activation, which may have a role in cognitive function regulation, results from various stimuli such as viruses, ER stress, and ROS (Hugon and Paquet, 2021; Costa-Mattioli and Walter, 2020; Zhu et al., 2011). We found that copper deposition in the brains of WD model mice can trigger a significant release of ROS, leading to oxidative stress damage. The excessive accumulation of copper in the brain, and its accompanying oxidative stress and PKR/eIF2α pathway activation, may contribute to the cognitive impairment observed in individuals with neurological WD. These findings indicate that the oxidative stress induced by copper overload might disrupt synaptic function by inihbiting the PKR/eIF2α pathway, ultimately causing cognitive impairment.

The hydroxyl free radical 8-OHdG is produced under conditions of oxidative stress or accumulation of oxygen-containing metabolites (Liu et al., 2017). Abnormal increases in this marker are associated with various neurodegenerative diseases, including AD and Parkinson’s disease (Sidorova and Domanskyi, 2020; Cao and Chen, 2020). According to the results of a previous study (Kamat et al., 2016), synaptic dysfunction may be caused by oxidative stress factors interacting with other molecules. In our study, we found associations between synaptic dysfunction, the PKR/eIF2α pathway and the pathophysiology and progression of WD. The effectiveness of C16 as a WD treatment has been confirmed *in vivo* using various disease models. In our study, the ability of C16 to prevent oxidative stress in the murine model of WD resulted in a reduction in the number of 8-OHdG –positive cells in the hippocampi of model mice. Metal ions deposited in various regions of the brain can lead to oxidative stress due to metabolic alterations. Synaptic function can be impaired by metal ion imbalances, oxidative stress, or both; and, it results in chronic neurodegeneration and cognitive deficits. The discovery that the most sensitive indicator of copper excess is a decrease in the ratio of GSH to GSSG is especially significant because it indicates an underlying neurodegenerative mechanism. We found that C16 inhibited the phosphorylation of PKR and the subsequent activation of the PKR/eIF2α pathway in the WD mouse model, all the while demonstrating a beneficial impact on synaptic functional indicators. Our results suggest that C16 attenuates synaptic dysfunction and prevents neuronal damage, cognitive impairment, and neurodegeneration in TX mice. In all, our findings should help clarify the neuropathological mechanisms of WD leading to cognitive impairment and they may be useful to scientists exploring treatment strategies.

## 5 Conclusion

**FIGURE 8:**
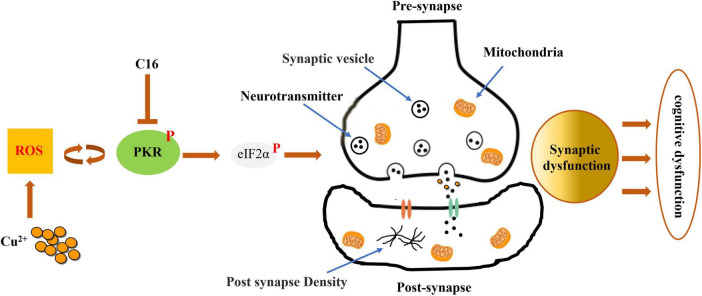
Inhibiting the activation of the PKR/eIF2α pathway attenuates the synaptic dysfunction caused by copper in murine model of Wilson’s disease (WD). By preventing the phosphorylation of PKR in WD, the PKR inhibitor C16 effectively inhibits the PKR/eIF2α pathway and attenuates synaptic dysfunction, safeguarding against neuronal damage, cognitive decline, and neurodegenerative disorders.

Our findings demonstrate that the PKR/eIF2α pathway and synaptic dysfunction have active roles in the development of WD (Figure 8). Additionally, we observed that inhibiting this pathway can prevent copper-induced synaptic dysfunction and cognitive impairment. Thus, PKR may be a promising pharmacological target for the treatment of copper-induced neuropathology in WD.

## Data Availability

The original contributions presented in this study are included in this article/supplementary material, further inquiries can be directed to the corresponding authors.

## References

[B1] An Y. Li S. Huang X. Chen X. Shan H. Zhang M. (2022). The role of copper homeostasis in brain disease. *Int. J. Mol. Sci.* 23:13850. 10.3390/ijms232213850 36430330 PMC9698384

[B2] Bach M. E. Hawkins R. D. Osman M. Kandel E. R. Mayford M. (1995). Impairment of spatial but not contextual memory in CaMKII mutant mice with a selective loss of hippocampal LTP in the range of the theta frequency. *Cell* 81 905–915. 10.1016/0092-8674(95)90010-1 7781067

[B3] Banci L. Bertini I. Cantini F. Ciofi-Baffoni S. (2010). Cellular copper distribution: A mechanistic systems biology approach. *Cell. Mol. Life Sci.* 67 2563–2589. 10.1007/s00018-010-0330-x 20333435 PMC11115773

[B4] Cao X. Chen P. (2020). Changes in serum amyloid A (SAA) and 8-OHdG in patients with senile early cognitive impairment. *Med. Sci. Monit.* 26:e919586. 10.12659/MSM.919586 32006457 PMC7009755

[B5] Cheng H. Zhang G. Guo B. Sun Y. Zhang G. Wang Y. (2021). T-006 improves learning and memory function and regulates synaptic associated protein expression in APP/PS1/Tau triple transgenic mice. *J. Sun Yat-sen Univ.* 42 667–675.

[B6] Chung W.-S. Welsh C. A. Barres B. A. Stevens B. (2015). Do glia drive synaptic and cognitive impairment in disease? *Nat. Neurosci.* 18 1539–1545. 10.1038/nn.4142 26505565 PMC4739631

[B7] Costa-Mattioli M. Walter P. (2020). The integrated stress response: From mechanism to disease. *Science* 368:eaat5314. 10.1126/science.aat5314 32327570 PMC8997189

[B8] Dong J. Xiang G. Xia X. Xu L. Wen P. Xu C. (2024). Aberrant copper metabolism and hepatic inflammation cause neurological manifestations in a mouse model of Wilson’s disease. *J. Neuroinflammation* 21:235. 10.1186/s12974-024-03178-5 39334421 PMC11437830

[B9] Gal-Ben-Ari S. Barrera I. Ehrlich M. Rosenblum K. (2019). PKR: A kinase to remember. *Front. Mol. Neurosci.* 11:480. 10.3389/fnmol.2018.00480 30686999 PMC6333748

[B10] Greenough M. A. Camakaris J. Bush A. I. (2013). Metal dyshomeostasis and oxidative stress in Alzheimer’s disease. *Neurochem. Int.* 62 540–555. 10.1016/j.neuint.2012.08.014 22982299

[B11] Hirata Y. Iwasaki T. Makimura Y. Okajima S. Oh-Hashi K. Takemori H. (2019). Inhibition of double-stranded RNA-dependent protein kinase prevents oxytosis and ferroptosis in mouse hippocampal HT22 cells. *Toxicology* 418 1–10. 10.1016/j.tox.2019.02.012 30817950

[B12] Hugon J. Paquet C. (2021). The PKR/P38/RIPK1 Signaling pathway as a therapeutic target in Alzheimer’s disease. *Int. J. Mol. Sci.* 22:3136. 10.3390/ijms22063136 33808629 PMC8003462

[B13] Hureau C. (2023). Can the level of copper in the hippocampus witness type-II diabetes versus Alzheimer’s disease? *EBiomedicine* 87:104403. 10.1016/j.ebiom.2022.104403 36516609 PMC9768223

[B14] Jomova K. Makova M. Alomar S. Y. Alwasel S. H. Nepovimova E. Kuca K. (2022). Essential metals in health and disease. *Chem. Biol. Interact.* 367:110173. 10.1016/j.cbi.2022.110173 36152810

[B15] Kamat P. K. Kalani A. Rai S. Swarnkar S. Tota S. Nath C. (2016). Mechanism of oxidative stress and synapse dysfunction in the pathogenesis of Alzheimer’s disease: Understanding the therapeutics strategies. *Mol. Neurobiol.* 53 648–661. 10.1007/s12035-014-9053-6 25511446 PMC4470891

[B16] Kumari P. Roy K. Wadhwa M. Chauhan G. Alam S. Kishore K. (2020). Fear memory is impaired in hypobaric hypoxia: Role of synaptic plasticity and neuro-modulators in limbic region. *Life Sci.* 254:117555. 10.1016/j.lfs.2020.117555 32188570

[B17] Liu Z. Liu Y. Tu X. Shen H. Qiu H. Chen H. (2017). High serum levels of malondialdehyde and 8-OHdG are both associated with early cognitive impairment in patients with acute ischaemic stroke. *Sci. Rep.* 7:9493. 10.1038/s41598-017-09988-3 28842715 PMC5573400

[B18] Ma Q. Ying M. Sui X. J. Zhang H. M. Huang H. Y. Yang L. Q. (2015). Chronic copper exposure causes spatial memory impairment, selective loss of hippocampal synaptic proteins, and activation of PKR/eIF2α pathway in mice. *J. Alzheimers Dis.* 43 1413–1427. 10.3233/JAD-140216 25159668

[B19] Maung M. T. Carlson A. Olea-Flores M. Elkhadragy L. Schachtschneider K. M. Navarro-Tito N. (2021). The molecular and cellular basis of copper dysregulation and its relationship with human pathologies. *FASEB J.* 35:e21810. 10.1096/fj.202100273RR 34390520

[B20] Mouton-Liger F. Paquet C. Dumurgier J. Bouras C. Pradier L. Gray F. (2012). Oxidative stress increases BACE1 protein levels through activation of the PKR-eIF2α pathway. *Biochim. Biophys. Acta* 1822 885–896. 10.1016/j.bbadis.2012.01.009 22306812

[B21] Pal A. Prasad R. (2015). An overview of various mammalian models to study chronic copper intoxication associated Alzheimer’s disease like pathology. *Biometals* 28 1–9. 10.1007/s10534-014-9799-3 25307560

[B22] Palmieri G. R. De Michele G. Matarazzo M. Di Dato F. Perillo S. Dello Iacovo D. C. P. (2022). Prevalence and features of non-motor symptoms in Wilson’s disease. *Parkinsonism Relat. Disord.* 95 103–106. 10.1016/j.parkreldis.2022.01.016 35093711

[B23] Rodriguez-Castro K. I. Hevia-Urrutia F. J. Sturniolo G. C. (2015). Wilson’s disease: A review of what we have learned. *World J. Hepatol.* 7 2859–2870. 10.4254/wjh.v7.i29.2859 26692151 PMC4678372

[B24] Sidorova Y. Domanskyi A. (2020). Detecting oxidative stress biomarkers in neurodegenerative disease models and patients. *Methods Protoc.* 3:66. 10.3390/mps3040066 32987935 PMC7712543

[B25] Tönnies E. Trushina E. (2017). Oxidative stress, synaptic dysfunction, and Alzheimer’s disease. *J. Alzheimers Dis.* 57 1105–1121. 10.3233/JAD-161088 28059794 PMC5409043

[B26] Tsang T. Davis C. I. Brady D. C. (2021). Copper biology. *Curr. Biol.* 31 R421–R427. 10.1016/j.cub.2021.03.054 33974864

[B27] Ulrich J. D. Finn M. B. Wang Y. Shen A. Mahan T. E. Jiang H. (2014). Altered microglial response to Aβ plaques in APPPS1-21 mice heterozygous for TREM2. *Mol. Neurodegener.* 9:20. 10.1186/1750-1326-9-20 24893973 PMC4049806

[B28] Xu D. Hou H. Wang X. Wang J. Cheng N. Wu J. (2015). Contents of Cu in copper overload rats’ liver, kidney and brain tissues and its influence on MT and Aβ. *Chin. Arch. Trad. Chin. Med.* 33 299–301.

[B29] Zhao J. Shi Q. Tian H. Li Y. Liu Y. Xu Z. (2021). TDMQ20, a specific copper chelator, reduces memory impairments in Alzheimer’s disease mouse models. *ACS Chem. Neurosci.* 12 140–149. 10.1021/acschemneuro.0c00621 33322892

[B30] Zhu P. J. Huang W. Kalikulov D. Yoo J. W. Placzek A. N. Stoica L. (2011). Suppression of PKR promotes network excitability and enhanced cognition by interferon-γ-mediated disinhibition. *Cell* 147 1384–1396. 10.1016/j.cell.2011.11.029 22153080 PMC3569515

